# Differences in physical activity and sedentary time in relation to weight in 8–9 year old children

**DOI:** 10.1186/1479-5868-5-67

**Published:** 2008-12-12

**Authors:** Lisa R Purslow, Claire Hill, Jenny Saxton, Kirsten Corder, Jane Wardle

**Affiliations:** 1 Department of Epidemiology and Public Health, University College London, London, UK; 2 Institute of Metabolic Science, MRC Epidemiology Unit, Cambridge, UK

## Abstract

**Background:**

The health benefits of physical activity for children are well established. Although objective measures of physical activity are increasingly used there is still a lack of adequate data on physical activity in children. Sex differences in physical activity have been consistently demonstrated and lower levels of physical activity in obese than non-obese children have been shown. However, differences across the whole weight spectrum have not been examined in detail. The aim of this study was to assess associations between physical activity and sedentary time across the weight spectrum in children, and to determine whether the associations differed by sex.

**Methods:**

Participants in the current study were 176 boys and 169 girls aged 8–9 years old taking part in a longitudinal study of associations between eating behaviours, physical activity and weight gain during childhood. Height, weight and waist circumference were measured, and physical activity data were collected using an Actigraph model GT1M worn for 5 consecutive days. Associations between sex, weight and physical activity were analysed using linear regression models.

**Results:**

Boys had higher total activity (mean difference = 119, p < 0.001) and more minutes of moderate and vigorous physical activity (MVPA) (mean difference = 25, p < 0.001) than girls. A higher percentage of boys (72%) than girls (30%) met current physical activity guidelines of 60 minutes MVPA per day. In boys, weight status significantly predicted total activity (p = 0.001) and MVPA (p = 0.001) but there were no significant associations in girls. There was no significant difference in time spent sedentary between boys and girls, and weight status did not predict sedentary time.

**Conclusion:**

In boys, physical activity was progressively lower across the weight spectrum, but in girls physical activity was consistently low across all weight categories. Intervention is required prior to 8 years old to prevent weight-related declines in physical activity in boys and further research is required to determine at what age, if ever, weight related differences in physical activity are apparent in girls.

## Background

The health benefits of physical activity are well established and for children they include avoidance of weight gain and hypertension, increased bone mineral density and improvement of mental health [1]. A recent review recommended that children should be active for at least 60 minutes a day at a moderate to vigorous intensity for optimal health benefits [2]. Although objective measures of physical activity are increasingly being used there is still a lack of data on children's physical activity [3] especially with regard to the association with weight status. Sedentary lifestyle patterns have been associated with obesity in children and adolescents [4]. A recent review of sedentary behaviour and obesity development in children concluded that while sedentary behaviours, especially TV viewing, are associated with obesity there is no evidence that sedentary behaviour displaces physical activity [5]. Several studies have also shown a negative association between body fat and vigorous physical activity [6-8] and total activity [9,10] as well as a positive association with TV viewing [11]. It is therefore important to consider associations between both physical activity and sedentary behaviour and weight [11].

Sex differences in children's physical activity are well established, with boys showing greater levels of total activity and moderate to vigorous intensity physical activity (MVPA) than girls from age 4 upwards [3,12-14]. Differences in physical activity have also been demonstrated between obese and non-obese adults [15] adolescents [6,16] and children [3,11,17,18], with obese individuals showing lower levels of physical activity. Sex-by-weight interactions have been observed in adults [15] and children [19], with large differences in physical activity between obese and non-obese males and small or no differences between obese and non-obese females. A recent review [3] alluded to sex differences in the association between physical activity and weight, but a number of studies have failed to find this effect [8,16,18].

Although it appears that physical activity is associated with weight, and that sex may moderate the association, existing studies are based on comparisons between obese cases and non-obese controls. Adiposity has an approximately normal distribution [20] and a behavioural susceptibility model of obesity has been proposed which conceives appetitive and activity traits as a continuum to explain variation across the continuum of weight [20,21]. Psychometric [20] and behavioural measures of eating traits that are know to be linked to obesity show a graded association across the weight spectrum [22]. We are not aware of any existing studies that have examined associations with physical activity in this way. As activity is a vital component of the energy balance equation, it is reasonable to assume that physical activity may also show graded associations across the weight spectrum rather than just being 'abnormal' in obese individuals.

Population level data on physical activity levels in children are still sparse, especially in the UK [3]. However, numerous studies have shown large declines in physical activity from late childhood to early adolescence especially in girls [23-25] and it has recently been suggested that the decline begins before the teenage years [26]. Therefore, the purpose of this study was to examine associations between activity/sedentary time and weight across the whole spectrum of adiposity in pre-adolescent children, and determine whether the associations differed by sex. Associations between activity/sedentary time and other measures of adiposity (body fat and waist circumference) were also examined.

## Methods

### Participants

Participants were children aged 8–9 years old taking part in the Physical Exercise and Appetite in CHildren Study (PEACHES), a longitudinal study of associations between eating behaviours, physical activity and weight gain during childhood. Invitation letters were sent to all parents of all children in Years 3 and 4 in five schools in London, UK (n = 531). At baseline, 400 children were weighed and measured. Anthropometric and physical activity data were collected from all participating children when they were in Year 4. Activity data were collected from 345 children (176 boys and 169 girls). The study was approved by the University College London Committee on the Ethics of Non-NHS Human Research.

### Measures

#### Socio-demographic data

Schools provided the date of birth, sex, postcode and ethnicity of participating children. Socioeconomic status (SES) was classified by area-level socioeconomic deprivation by matching postcodes to 1991 census data to derive Townsend scores [27]. For dwellings built after 1991 and where postcodes were missing the average Townsend score for the school was used. Due to small numbers in each of the wide variety of non-white ethnic groups, children were classified as 'white' or 'non-white' for analysis.

#### Anthropometry

Trained researchers weighed and measured children using standardised protocols. Height was measured in bare feet using a Leicester height measure (Seca, Birmingham, UK) and recorded to the nearest millimetre. Weight and fat mass were measured in light indoor clothing using the Tanita TBF-300MA Body Composition Analyser (Tanita Corporation, Tokyo, Japan) and recorded to the nearest tenth of a kilogram. Waist circumference was measured over a single layer of clothing 4 cm above the navel using Seca 200 circumference measuring tape [28]. Inter-rater reliability of anthropometric measurements was calculated in a subsample of children (n = 30) and high correlations of 1.0, 0.99, 0.99, 0.89 were found for weight, fat mass, height and waist circumference respectively.

#### Physical Activity

Children were asked to wear an Actigraph model GT1M for 5 consecutive days including 2 weekend days. The Actigraph (formerly CSA/MTI) is the most commonly used accelerometer in physical activity research [29] and has been validated for use in children [30,31]. The monitors were set to record in one minute epochs and were worn on an elastic belt with the monitor placed on the right hip during waking hours and only removed for bathing, showering and swimming. To maximise compliance two level incentive schemes were employed: a prize draw and a girl/boy competition. Each child who wore their monitor every day was awarded a ticket for a prize draw to win a £25 gift voucher. In addition, accelerometers were personalised using cartoon character stickers to create Team Angel (girls) and Team Arnie (boys) and each child in the team with the highest compliance received a £5 gift voucher.

### Data treatment and statistical analysis

BMI was calculated as weight (kg)/height (m^2^). BMI and waist circumferences were transformed into age- and gender-appropriate BMI sd and waist sd scores based on the British 1990 reference data [32] using the Imsgrowth macro (from ). The same program was used to group children according to the International Obesity Taskforce (IOTF) cut-offs for healthy-weight, overweight, and obesity [33] and the recently proposed criteria for underweight (thinness grade 1, 2 or 3) [34]. Due to small numbers in the obese category, overweight and obese groups were combined. In addition, the healthy weight category was subdivided into lower-healthy-weight (50^th ^centile or under but not meeting criteria for underweight) and higher-healthy-weight (above 50^th ^centile but not meeting criteria for overweight) to examine the distribution of physical activity across the adiposity continuum. Fat mass index (FMI) [35] was calculated by dividing fat mass (kg) by height (m^1.28^) to adjust for body size. The optimal power to raise height (1.28) was derived from the data so that the relationship between fat mass and height was removed [36].

Actigraph files were processed using a custom written programme (MAHUffe: ). Time periods of at least 10 minutes of continuous zeros were considered to represent removal of the monitor and were excluded [37,38] and only days with a registered time of at least 600 minutes were included for analysis. Visual inspection of the data indicated that while some children had removed the monitor at night others continued to wear the monitor for 24 hours. Therefore, only data recorded between 7 am – 9 pm were analysed in order to allow comparability between children. Total physical activity was calculated as the average accelerometer counts/min over the full period of valid recording. The amount of time spent sedentary (< 100 counts/min), and in light (100–1999 counts/min), moderate (2000–3999 counts/min), vigorous (4000–6999 counts/min) and very vigorous (> 7000 counts/min) activity were calculated per valid day. A cut-off of 2000 cpm has been used previously in a large sample of 9 year olds to indicate time spent in at least moderate activity [39]. Moderate and vigorous physical activity (MVPA) was calculated as the sum of moderate, vigorous and very vigorous activity per day. Previous research has shown that 3 days of at least 600 minutes gave reliability and power > 90% [40], and therefore only children with at least 3 days (including at least 1 weekend day) of valid data were included for analyses.

Analyses were carried out using SPSS (Version 14; SPSS Inc, Chicago, IL). Linear trends were used to examine physical activity across the weight spectrum. As the distribution of weight in our sample was representative of the UK population (HSE, 2006; data publicly available from the UK Data Archive: ) the weighted linear term was appropriate. We used linear regression analyses to assess the relations between BMI sd score and physical activity. Sex differences in physical activity (total and MVPA) have been widely reported and significant sex by BMI interactions were apparent in our data. Therefore all analyses were stratified by sex. We examined whether the association between either BMI sd score, fat mass index or waist sd score and physical activity were modified by area-based deprivation or ethnicity. Similar analyses were also carried out for time spent sedentary although there were no significant sex by BMI interactions so combined results are presented. The intraclass correlation between school and total physical activity, MVPA and sedentary time were below the conventional value of 0.05 and the number of clusters was small (5), therefore correction for clustering was not appropriate [41].

## Results

Compliance (at least 3 days of 600 minutes or more of valid data) was achieved by 301 (88%) participants. The mean number of days the monitor was worn for those included was 4, and in each subgroup the mean was also 4. For the majority of children (257) the monitor was worn for 2 week days and 2 weekend days. There were no differences in age, sex, BMI sd score, SES or ethnicity between children who did and did not achieve full compliance. Total activity (counts/min) and minutes of MVPA were significantly higher in children who provided less than 3 days of valid data compared to those who provided 3 or more days of valid data (t = 3.03, df = 338, p = 0.003, effect size = 0.52 and t = 2.75, df = 338, p = 0.006, effect size = 0.47 respectively).

Descriptive statistics and physical activity data are shown in table 1. Boys had higher levels of total activity (t = 7.2, df = 299, p < 0.001) and MVPA (t = 8.8, df = 267, p < 0.001) than girls. A higher percentage of boys met current physical activity guidelines of 60 minutes MVPA per day. Descriptive statistics and physical activity levels for each weight category are shown in table 2.

**Table 1:** Descriptive statistics and main physical activity variables

	All (n = 301)	Boys (n = 155)	Girls (n = 146)	P value^¥^
Age (years)	8.6 (0.4)	8.7 (0.4)	8.8 (0.6)	> 0.05

Height (cm)	134.0 (6.7)	134.2 (6.8)	133.9 (6.7)	> 0.05

Weight (kg)	30.5 (6.9)	30.7 (7.0)	30.3 (6.8)	> 0.05

BMI SDS*	0.15 (1.32)	0.20 (1.38)	0.09 (1.25)	> 0.05

Waist SDS^±^	0.87 (1.04)	0.97 (1.05)	0.77 (1.02)	> 0.05

Fat mass index (kg/m^1.28^)	4.4 (3.0)	4.2 (3.0)	4.6 (3.0)	> 0.05

Socioeconomic status^◇^	4 (4)	4 (3)	4 (3)	> 0.05

Ethnicity^≠^	46	48	44	> 0.05^v^

Total activity (counts/min)	606 (154)	663 (150)	544 (134)	< 0.001

Sedentary activity^$ ^(min/day)	330 (119)	321 (109)	338 (128)	> 0.05

MVPA^+ ^(min/day)	66 (28)	78 (29)	53 (19)	< 0.001

Percentage meeting recommended activity level^∞^	52	72	30	< 0.001^v^

All values are mean (SD) unless otherwise stated

*Body mass index standard deviation score

^± ^Waist standard deviation score

^◇^Measured using the Townsend index score with imputed values from school postcode, UK average is zero with positive score representing higher than average deprivation

^≠^Percentage white

^$^Cut points used: Sedentary < 100; Light 100–1999; Moderate 2000–3999; Vigorous 4000–6999; Very Vigorous 7000+

^+^Moderate to vigorous physical activity

^∞^Recommended activity level ≥ 60 min of MVPA activity daily

^¥ ^Independent samples T-test

^√^Chi squared test

**Table 2:** Descriptive statistics and physical activity levels by weight group

	Thinness grade 1 or 2 (n = 44)	Lower normal weight (n = 96)	Higher normal weight (n = 102)	Overweight and Obese (n = 59)	P for trend*
Height (cm)	130.8 (6.5)	132.2 (6.4)	134.5 (6.3)	138.6 (5.6)	< 0.001

Weight (kg)	23.4 (2.5)	26.4 (2.7)	31.3 (3.4)	41.0 (5.3)	< 0.001

BMI SDS*	-1.81 (0.47)	-0.65 (0.36)	0.63 (0.40)	2.1 (0.54)	< 0.001

Waist SDS^±^	-0.32 (0.65)	0.28 (0.55)	1.09 (0.55)	2.35 (0.55)	< 0.001

Fat mass index (kg/m^1.28^)	2.1 (1.6)	2.8 (1.4)	4.3 (1.5)	8.9 (3.1)	< 0.001

Overall activity (counts/min)	651 (179)	616 (173)	590 (135)	582 (127)	0.013

Light activity (min/day)^$^	364 (62)	358 (56)	366 (58)	390 (49)	0.006

Moderate activity (min/day)^$^	59 (21)	55 (23)	53 (21)	52 (18)	0.085

Vigorous activity (min/day)^$^	14 (12)	12 (11)	10(8)	7 (5)	< 0.001

Very vigorous activity (min/day)^$^	2 (2)	1 (2)	1 (2)	0.3 (0.6)	< 0.001

Sedentary activity^$ ^(min/day)	314 (52)	327 (89)	325 (128)	353 (169)	0.146

MVPA^+ ^(min/day)	74 (31)	68 (31)	64 (26)	59 (21)	0.002

Meeting recommended activity level (%)^∞^	68	55	47	42	0.042^v^

All values are mean (SD) unless otherwise stated

* Oneway ANOVA with 4 weight groups used to calculate p for linear trend

^$^Cut points used: Sedentary < 100; Light 100–1999; Moderate 2000–3999; Vigorous 4000–6999; Very Vigorous 7000+

^+^Moderate to vigorous physical activity

^∞^Recommended activity level ≥ 60 min of MVPA activity daily

^√^Chi squared test

**Figure 1:**
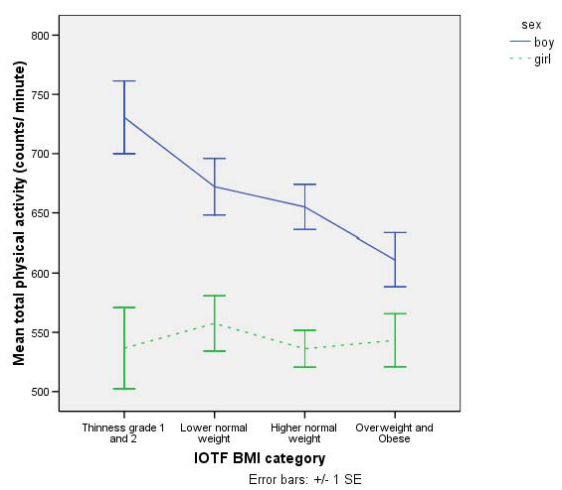
Total physical activity ± SE by International Obesity Task Force (IOTF) Body Mass Index (BMI) category for girls and boys. Girls are in dashed lines and boys are represented by continuous line. A significant p-value (0.002) for trend was observed in boys but not girls.

We examined physical activity across the weight spectrum in boys and girls (figure 1). For boys total activity and MVPA were progressively lower from thin to overweight/obese groups, with a significant linear trend for total activity (p = 0.002) and MVPA (p = 0.002). Linear regression models with weight as a continuous variable and adjusted for SES and ethnicity were also significant for total activity (β = -0.26, p = 0.002) and MVPA (β = -0.28, p = 0.001). For girls there was no significant linear trend and the regression models were not significant for either total activity or MVPA.

**Figure 2:**
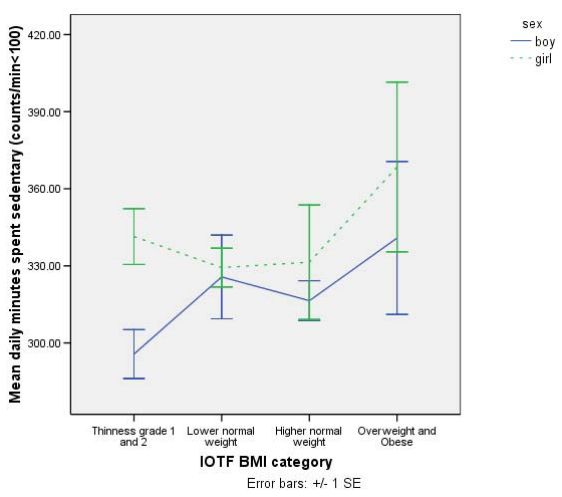
Time spent sedentary ± SE by International Obesity Task Force (IOTF) Body Mass Index (BMI) category for girls and boys. Girls are in dashed lines and boys are represented by continuous line. There was no significant trend for boys or girls.

Figure 2 shows time spent sedentary for boys and girls. There was no significant difference in sedentary time between boys and girls and no significant linear trend across the weight spectrum. There was no significant sex-by-BMI interaction so data were combined for regression analyses. A linear regression model adjusted for sex, SES and ethnicity was not significant.

Similar results were obtained when adiposity was indexed by fat mass index or waist sd score. All models were adjusted for SES and ethnicity. Analyses were done separately by sex because of the significant sex by BMI interactions. There was a significant association between fat mass index and total physical activity (β = -0.27, p = 0.001) and MVPA (β = -0.31, p < 0.001) in boys but no associations in girls. There was a significant association between waist sd score and total physical activity (β = -0.26, p = 0.002) and MVPA (β = -0.31, p < 0.001) in boys but no associations in girls.

There were no significant interactions with sex in models of sedentary time, therefore all models were run on both sexes combined and adjusted for age, sex, SES and ethnicity. There was a significant positive association between fat mass index and sedentary time (β = 0.18, p = 0.002), but no significant association between waist sd and sedentary time.

## Discussion

The purpose of this study was to characterise physical activity in children aged 8–9 years old and examine associations with sex and weight status. As has been demonstrated in children from 4 years upwards [12,13] boys had higher total activity and MVPA than girls. More than twice the number of boys (72%) than girls (30%) met the current UK children's physical activity guidelines of 60 minutes MVPA per day [2]. In addition to sex differences in physical activity, there were differences between the sexes in the association between physical activity and weight. In boys there was a graded decrease in total physical activity and MVPA from thinness grade 1 and 2 through the lower and higher healthy weight groups to overweight/obesity, whereas no weight-related differences in physical activity were seen in girls. In contrast, no sex differences were seen in sedentary time and fat mass index was significantly associated with sedentary time in both boys and girls.

No effects of socioeconomic position or ethnicity were seen in this study. Two previous UK studies have also shown no effect of socioeconomic position on physical activity or sedentary behaviour [3,42].

Total activity (counts/min) and minutes of MVPA were significantly higher in children who provided less than 3 days of valid data compared with those who provided 3 or more days of valid data. A small measurement effect of wearing the monitor has been reported previously [3,40] which may account for the higher levels of activity being recorded in children with fewer days of data.

Higher levels of physical activity in non-obese than obese children have been demonstrated [3,6,11,15-18] and our results confirm previous findings in adults [15] and children [19] of differential effects of sex by weight. However, we believe this is the first study to show sex differences in the association between physical activity and weight status across the whole weight spectrum. The graded negative association between physical activity and weight status in boys is consistent with the theory that physical activity can be an important contributing factor to the development of obesity [43]. However, the lack of association in girls warrants further investigation. It has previously been suggested that very low levels of physical activity in obese or non-obese girls masks differences by weight status [19] and our data confirm very low levels of activity in girls across the weight spectrum. Coupled with the small number of girls meeting current physical activity guidelines, these findings indicate that physical activity promotion in girls should start at a very young age to prevent the universally low levels of activity that are already present by age 8 years. It is worth noting that a recent longitudinal study in children aged from 5 to 8 years showed no changes in physical activity over time in either boys or girls [44]. This may indicate that the lack of physical activity in girls observed in the current study at age 8 would have already been present at age 5. It is therefore important to determine when, if ever, they show a graded association between physical activity and weight.

It is worth noting that the highest level of physical activity in our study was seen in boys in the thinness grade 1 and 2 categories which some may consider as an unhealthy weight. However, the IOTF thinness grades 1, 2 and 3 cut-points used in this study were only published in 2007 [34] and previously there was no guideline for clinical underweight in children. Thinness grades 1, 2 and 3 are equivalent to BMI at age 18 of 18.5, 17 and 16 respectively [34]. We had no children in grade 3 (the most severely underweight category) and only 7 children in grade 2. Therefore, the majority of children in the lowest weight category were in grade 1 and would not be considered clinically underweight. It is likely that previous research showing adverse health behaviours and outcomes associated with thinness referred to clinically underweight or failing-to-thrive children.

Associations between physical activity and the two other indices of adiposity (fat mass index and waist circumference) were remarkably similar to those with BMI sd score. In boys there was a negative association between physical activity and both fat mass index and waist circumference whereas no significant associations were seen in girls. Previous studies have shown that vigorous physical activity [6-8] and total activity [9,10] are negatively associated with body fat but these studies did not examine the results separately by sex. Our results support the existence of this relationship in boys but not girls. Given the apparently robust findings of little or no association between physical activity and weight in girls from the current and previous [3,19] studies, this suggests that studies using combined sex samples may have missed the differential associations.

We found no significant associations between sedentary activity and weight or waist circumference, but there was a significant positive association between sedentary time and fat mass index in boys. Although previous studies of sedentary activities and obesity have been equivocal [5], TV viewing has been independently associated with body fatness [5,11]. We have no way of knowing the types of sedentary behaviour our participants were involved in but future research should attempt to determine the specific association between different types of sedentary behaviours and measures of adiposity. This finding highlights the importance of assessing both physical activity and sedentary behaviour as high levels of both can occur within individuals, as seen in thinness grade 1 and 2 boys in the current study.

This study benefited from an ethnically and socio-economically diverse sample, which contributes to the generalisability of the findings. However, the cross-sectional nature of the study means that it is not possible to determine causal effects, i.e., whether low physical activity or high levels of sedentary behaviour lead to higher adiposity, or higher adiposity leads to decreased activity or increased sedentary time. A further limitation of the current study is that accelerometers are not able to capture swimming, cycling or load-bearing activities. Additionally, although 3 days of recording of at least 600 minutes gave reasonable reliability and power [40], it may not fully represent the individual's true activity. However, although this might weaken the association between physical activity and weight, the error introduced is random and should not introduce bias to the findings [39]. It has been suggested that epochs of 1-minute are not sufficiently sensitive to accurately reflect children's short bursts of activity [45] and our results may therefore underestimate time spent in MVPA. However, associations between sex and weight and MVPA were remarkably similar to the associations with total activity, which should not be susceptible to this measurement error.

## Conclusion

In summary we have demonstrated sex differences in the association between physical activity and adiposity. In boys there was a negative association between physical activity and weight status, no such association was seen in girls. Previous research has found that obese boys are less active than non-obese boys. The current findings indicate that rather than there being a threshold of weight above which physical activity declines, there is a continuous negative association between physical activity and weight in boys. The lack of a graded association in girls may be because all girls in this sample were very inactive and further research is required to determine at what age, if ever, weight-related differences in physical activity are apparent in girls.

## Competing interests

The authors declare that they have no competing interests.

## Authors' contributions

JW and CH devised the PEACHES study. LP, KC and JS prepared the data for analysis. LP devised the current study, analysed the data and drafted the manuscript. All authors contributed to the final draft of the manuscript.
